# The Effect of Temporal Perception on Weight Perception

**DOI:** 10.3389/fpsyg.2013.00040

**Published:** 2013-02-28

**Authors:** Hiroyuki Kambara, Duk Shin, Toshihiro Kawase, Natsue Yoshimura, Katsuhito Akahane, Makoto Sato, Yasuharu Koike

**Affiliations:** ^1^Precision and Intelligence Laboratory, Tokyo Institute of TechnologyYokohama, Japan; ^2^Department of Computational Intelligence and System Science, Tokyo Institute of TechnologyYokohama, Japan; ^3^CREST, Japan Science and Technology AgencyTokyo, Japan

**Keywords:** force illusion, weight perception, temporal perception, visuo-motor control, motor adaptation, temporal adaptation

## Abstract

A successful catch of a falling ball requires an accurate estimation of the timing for when the ball hits the hand. In a previous experiment in which participants performed ball-catching task in virtual reality environment, we accidentally found that the weight of a falling ball was perceived differently when the timing of ball load force to the hand was shifted from the timing expected from visual information. Although it is well known that spatial information of an object, such as size, can easily deceive our perception of its heaviness, the relationship between temporal information and perceived heaviness is still not clear. In this study, we investigated the effect of temporal factors on weight perception. We conducted ball-catching experiments in a virtual environment where the timing of load force exertion was shifted away from the visual contact timing (i.e., time when the ball hit the hand in the display). We found that the ball was perceived heavier when force was applied earlier than visual contact and lighter when force was applied after visual contact. We also conducted additional experiments in which participants were conditioned to one of two constant time offsets prior to testing weight perception. After performing ball-catching trials with 60 ms advanced or delayed load force exertion, participants’ subjective judgment on the simultaneity of visual contact and force exertion changed, reflecting a shift in perception of time offset. In addition, timing of catching motion initiation relative to visual contact changed, reflecting a shift in estimation of force timing. We also found that participants began to perceive the ball as lighter after conditioning to 60 ms advanced offset and heavier after the 60 ms delayed offset. These results suggest that perceived heaviness depends not on the actual time offset between force exertion and visual contact but on the subjectively perceived time offset between them and/or estimation error in force timing.

## 1 Introduction

The perception of an object’s heaviness is not only dependent on its weight. It is well known that information about the size of objects can mislead judgments of their heaviness. Charpentier (1891) was the first to demonstrate the size-weight illusion, where, given two objects of equal weight, the smaller object is perceived heavier than the larger one. In addition, the material and color of objects can also elicit the illusion of the weight difference (Wolfe, 1898; Seashore, 1899; De Camp, 1917). Although many studies have been performed in the attempt to understand the neural mechanisms underlying these illusions (Ross, 1969; Flanagan and Beltzner, 2000; Kim et al., 2002; Koike et al., 2006; Brayanov and Smith, 2010), we still lack a strong explanation of why these illusions occur (Ernst, 2009). However, taking into account that those illusions never occur when we lift objects with closing our eyes, it is clear that visual information affects weight perception.

In addition to visual information, timing of haptic sensation also seems to affect weight perception. To catch a ball falling from above, it is necessary to estimate the time when the ball will contact the hand in order to generate counteracting force at an appropriate timing (Lacquaniti and Maioli, 1989). Through a series of experiments in which participants caught a falling ball in a virtual reality environment (Hong et al., 2005; Kambara et al., 2011; Kawase et al., 2012), we investigated the neural mechanism underlying the estimation of time-to-contact (TTC), that is, the time remaining before contact. During these experiments, some participants happened to report that the weight of the falling ball was perceived differently when their TTC estimations were incorrect, even though the magnitude of force exerted on the hand was constant. From past studies (Ross, 1969; Davis and Roberts, 1976; Diedrichsen et al., 2007), it has been suggested that the misperception of force is caused by the mismatch between predicted sensory consequences of self-produced action and actual sensory inflow. However, it is not clear how error in estimating the timing of force affects perception of its magnitude.

In this study, we investigated the effect of temporal information on weight perception in a ball-catching task. We implemented a virtual reality environment where a falling ball was displayed on a large screen, and its load force was applied with a haptic device. To impose errors in the temporal estimates, the load force timing was advanced or delayed with respect to visual contact timing, i.e., the time when the ball made contact with the hand in the display. We then analyzed the relationship between perceived weight and time offset between load force exertion and visual contact. In addition, we also tested whether weight perception changes as the subjective judgment of simultaneity between visual and haptic stimuli changes. In our previous experiment (Kawase et al., 2012), we showed that participants’ judgment of temporal simultaneity was modified by experiencing tens of ball-catching trials in which the load force was applied with constant shifts from the time of visual contact. For example, when participants were persistently exposed to a 60 ms lag in load force timing, the point of subjective simultaneity (the relative time offset between visual and haptic stimuli at which the two stimuli are perceived as simultaneous) shifted in the direction of exposed lag. The shift in point of subjective simultaneity between visual and haptic stimuli has been observed in many other studies (Cunningham et al., 2001; Haggard et al., 2002; Stetson et al., 2006; Hanson et al., 2008; Harrar and Harris, 2008; Heron et al., 2009; Sugano et al., 2010). Recent studies have also started to focus on how motor learning is affected by adapted temporal perception (Tanaka et al., 2011; Honda et al., 2012). Here we investigated the effects of temporal conditioning on weight perception and its role in the sensory-motor process. Note that the two previous studies (Tanaka et al., 2011; Honda et al., 2012) investigated the effect of temporal adaptation only in one way. In other words, haptic stimulus always preceded visual stimulus in their studies. Our experimental design, on the other hand, allowed us to investigate how weight perception will be changed after conditioned not only to the situation where haptic precedes visual stimulus but also to the situation where haptic follows visual stimulus.

## 2 Materials and Methods

### 2.1 Participants

Six right-handed male adults (age: 21–39) took part in all experiments. All participants were recruited from within our institute. Participants had normal or corrected to normal vision, and had no cognitive or motor disorders. Experiments were performed in conformance with the Declaration of Helsinki on the use of human subjects in research, and written informed consent was obtained from all participants. The experimental procedures were approved by the ethics committee of the Tokyo Institute of Technology.

### 2.2 Experimental apparatus

**Figure 1:**
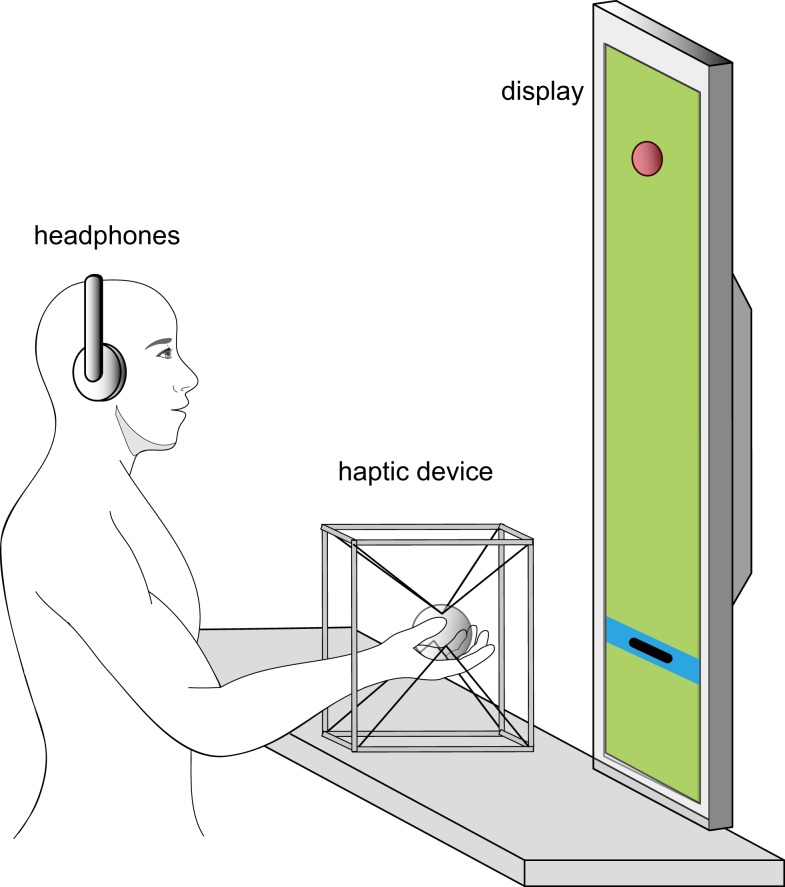
**Experiment system**. A virtual red ball (radius = 2 cm) and a black square cursor (width = 10 cm, height = 2 cm) projecting the hand position in the vertical direction were displayed on a plasma display. Subjects held a ball-shaped plastic grip attached to a “SPIDAR” haptic device, which consisted of eight motors and strings. Load force was applied through tension in the strings by the motors. The grip position was calculated from angle encoders attached to the motors. Subjects wore a pair of noise-canceling headphones to reduce the sound generated by the motors of the haptic device.

We implemented a virtual reality system for ball-catching tasks in order to manipulate the magnitude and timing of load force (Figure 1). The system was composed of a large plasma display (PDP503-CMX, 50 in, Pioneer) and a haptic device named “SPIDAR-G” (Kim et al., 2000; Akahane et al., 2006). This haptic device consists of eight motors (RE-max24, DC motor, Maxon) and eight strings attached to a ball-shaped grip. Force in a total of 6 degrees of freedom (DOF; 3DOF translational and 3DOF rotational) is generated by the strings whose tensions are controlled by the motors. In addition, the position of the center of the grip can be computed from the length of each string measured by encoders attached to the motors. The control and measurement frequency were both set as 1 ms.

During the experiments, participants sat on a chair in front of a table and display (Figure 1). Their shoulders were fixed to the back of the chair by means of a shoulder harness. We asked them to put their right forearms on the table and grasp the haptic grip with a supinated posture (palm up). Participants wore noise-canceling headphones (QuietComfort 3, BOSE) to reduce influence by external sounds (e.g., the sound of the motors).

A virtual ball was displayed on the screen as a red sphere of 2 cm radius and fell with 0 m/s initial velocity and 9.8 m/s^2^ acceleration. A black cursor in the shape of a 10 cm width and 2 cm height rectangle, projecting the hand position, moved in the vertical direction in accordance with the grip’s vertical position. After contacting with the black cursor, the ball would remain in contact and move along with the cursor. A blue bar of 3 cm height was also displayed as the reference area for initial hand position in ball-catching trials.

The load force of the ball was simulated by applying a downward force to the hand with SPIDAR-G. The duration of the load force was set to 1 s for the entire experiment. In addition, the load force was kept constant during that 1 s. However, the magnitude of the load force was modulated according to experiment conditions described later. We also induced time offsets in load force exertion with respect to visual contact. Here we use the term “time offset” to indicate relative time difference between when the load force was applied to the hand and when the ball contacted the upper edge of the black cursor in the display. Note that the vertical position where the ball contacts the black cursor changes trial by trial because ball-catching motion includes some variant amount of hand movement. Therefore, we cannot know the exact timing of visual contact prior to the moment it actually happens. In the case where force is required to precede visual contact, it is impossible to make the actual time offset match exactly with the intended time offset. To make them as close as possible, TTC, time remaining before contact, was computed at every time step with respect to the current position of the black cursor. Then the force was applied at the time when TTC fell below the intended offset. Here, TTC at time *t* is computed as

(1) $$TTC\left(t\right)=\sqrt{\frac{2\left(h_{0}-x_{t}\right)}{g}}-t,$$

where *t* is the elapsed time from when the ball starts to fall, *g* is the gravitational acceleration, and *h*_0_ and *x_t_* are the initial height of the ball and position of the black cursor at time *t*, respectively. After completing all experiments, we verified how much the actual offset deviated from the intended in all trials where force was designed to precede visual contact. The largest deviation was 5.0 ms for a trial where force was supposed to be applied 120 ms before visual contact. The mean value was 1.3 ms. With the smallest time offset used in the experiments being 15 ms, these unavoidable force timing errors appeared to be negligible.

### 2.3 Experimental protocol

Participants underwent three experiments 1, 2, and 3, conducted on separate days. All participants performed Experiment 1 first. The order of Experiments 2 and 3 was randomized among all participants, with half performing Experiment 2 before 3 and the other half performing Experiment 2 after 3.

Each experiment was organized into three sessions “Conditioning,” “Simultaneity Test,” and “Weight Perception Test” sessions, presented in this order. Rest breaks of several minutes were taken between sessions.

Experiment 1 was conducted to investigate how the time offset between load force exertion and visual contact affects perceived weight of the ball. In the Conditioning session of Experiment 1, participants performed tens of ball-catching trials where the load force was applied simultaneously with visual contact. Then in the Weight Perception Test session, unpredictable time offsets were induced during ball-catching trials to investigate how participants’ perception of the ball’s weight changed with respect to time offset. In Experiments 2 and 3, we investigated how weight perception changes after participants were persistently exposed to constant offsets given during the Conditioning sessions. The load force was persistently applied in advance to visual contact in Experiment 2, and delayed from visual contact in Experiment 3. The Weight Perception Test sessions in Experiments 2 and 3 were conducted to test how the ball’s weight was perceived when the same offset as that used in Experiment 1 was applied.

**Figure 2:**
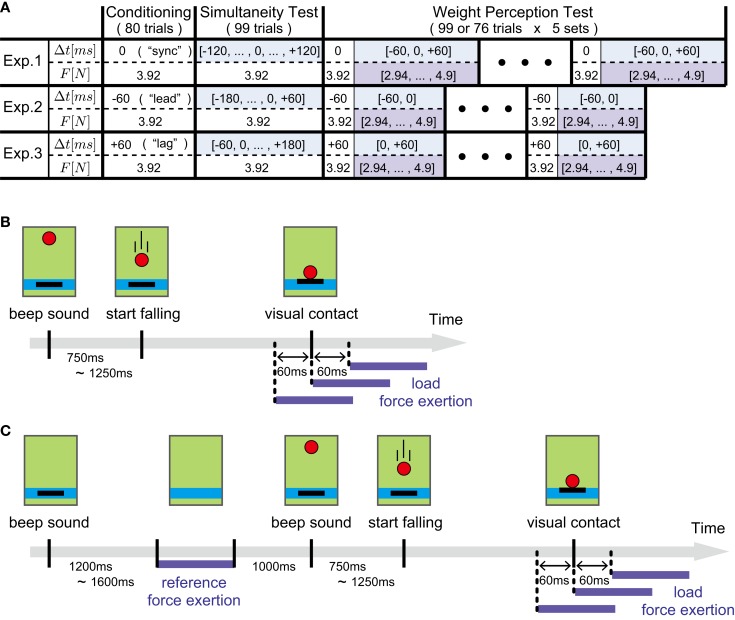
**Experimental protocol**. **(A)** An outline of the experimental protocol of the three experiments. Time offset Δ*t* and load force magnitude *F* in each trial are shown for the three sessions, i.e., conditioning, simultaneity test, and weight perception test sessions. Δ*t* and *F* shown with color backgrounds indicate that they are chosen randomly in each trial. The details of their values are list in Tables 1 and 2. **(B)** The sequence of events in a single ball-catching trial during the Conditioning session. The load force is applied at the same time (Experiment 1), 60 ms before (Experiment 2), or 60 ms after (Experiment 3) the ball contacts the hand cursor in the display. **(C)** The sequence of events in a single weight judgment trial during the Weight Perception Test session. After applying the constant magnitude reference force, participants performed ball-catching. The timing of load force exertion was chosen randomly from three candidates corresponding to the times simultaneous to (0 ms offset trials), 60 ms before (−60 ms offset trials), or 60 ms after (+60 ms offset trials) visual contact of the ball.

An outline of the experimental protocol is illustrated in Figure 2A. In the following, we describe in detail the protocols of the three sessions of each experiment.

#### 2.3.1 Conditioning session

In the Conditioning session, each participant performed 80 ball-catching trials. The inter-trial interval was 2 s. The sequence of events in a single trial is shown in Figure 2B. Before the start of each trial, participants were asked to set the black cursor inside the blue bar indicating the initial hand position. At the beginning of the trial, the ball appeared at 80 cm above the blue bar, accompanied with a beep sound. The ball started falling with a random delay ranging from 0.75 to 1.25 s. The ball load force was then applied with or without time offset from visual contact. In Experiment 1, the load force was synchronized with visual contact. In Experiment 2, the force was applied 60 ms before visual contact, and in Experiment 3, 60 ms after visual contact. The magnitude of load force was 3.92 N for all Conditioning sessions. This value was selected to simulate the feeling of catching a ball weighing 400 g. In accordance with the time offsets used in the three experiments, we denoted the Conditioning sessions as “sync” (Experiment 1), “lead” (Experiment 2), and “lag” (Experiment 3).

We instructed participants to counteract the load force so as to keep the black cursor within the blue bar as consistently as possible. We also asked them to avoid co-contracting antagonistic muscle pairs of the forearm and try to generate upward force against the load force.

#### 2.3.2 Simultaneity test session

In the Simultaneity Test session, each participant underwent 99 ball-catching trials. The sequence of the events in a single trial was the same as that shown in Figure 2B, except for the time offset values. The list of offsets used in each of the three experiments is shown in Table 1 with their appearance frequencies within the session. The offset for each trial was selected randomly from the list. The initial height of the ball was also selected randomly from one of the following heights, 70, 80, or 90 cm. Appearance frequencies of the three heights were equal to each other. The magnitude and the duration of load force in each trial were 3.92 N and 1 s, respectively irrespective of the initial heights.

**Table 1:** **Time offset values and frequencies in the Simultaneity Test session**.

Experiment # (conditioning type)	Time offset [ms] (appearance frequency within the session)								
Experiment 1 (“sync”)	−120 (6)	−60 (12)	−30 (12)	−15 (12)	0 (15)	15 (12)	30 (12)	60 (12)	120 (12)
Experiment 2 (“lead”)	−180 (6)	−120 (12)	−90 (12)	−75 (12)	−60 (15)	−45 (12)	−30 (12)	0 (12)	60 (12)
Experiment 3 (“lag”)	−60 (6)	0 (12)	30 (12)	45 (12)	60 (15)	75 (12)	90 (12)	120 (12)	180 (12)

*Negative or positive offset values indicate that force exertion preceded or followed visual contact, respectively*.

Just as in the Conditioning session, we asked the participants to counteract the load force to catch the virtual ball. The participants were instructed to make judgments about the temporal order of visual contact and force exertion. We asked them to report which event occurred first by pressing the left or right button of a computer mouse held in their left hands. Time for judgment was not restricted and a successive trial started 2 s after either button was pressed. Note that the reason why the initial height of the ball was changed trial by trial is to force participants to make the temporal order judgment purely from visual and haptic sensations. If we fix the initial height, the duration of ball’s movement, time required the ball to reach the hand in the display, becomes the same in every trial. It might be possible that the participants learn the timing of visual contact and compare the timing of haptic sensation with that learned timing. Therefore, the initial height was determined randomly in each trial in the Simultaneity Test session.

#### 2.3.3 Weight perception test session

In each of the three experiments, the Weight Perception Test session was organized into five sets. A single set was composed of 99 trials in Experiment 1, and 76 trials in Experiment 2 and 3. Rest breaks of several minutes were taken between sets. In each set, participants first performed 30 ball-catching trials without any perceptual judgment. The time offsets for the first 30 ball-catching trials were the same as those in the Conditioning session in each experiment. The rest of the trials were weight judgment trials in which participants were asked to compare the heaviness of the ball and a reference force. Figure 2C shows the time sequence of the events in a single weight judgment trial. First, participants were asked to set the black cursor inside the blue bar. Then a beep sound was generated signifying the beginning of the trial. At the same time, the black cursor disappeared from the display. After a random delay ranging from 1.2 to 1.6 s, a reference force with magnitude 3.92 N was applied for 1 s. After the 1 s time interval, the ball and the black cursor appeared with a second beep sound. Participants then performed the ball-catching task after a random delay ranging from 0.75 to 1.25 s. Time offset was again imposed between visual contact and load force exertion. Its value was selected randomly from one of the following values; −60, 0, or +60 ms in Experiment 1, −60 or 0 ms in Experiment 2, and 0 or +60 ms in Experiment 3. Here, negative or positive offset signs indicate that load force preceded or followed visual contact, respectively. Each offset appeared 23 times in every set. Note that we did not include +60 ms offset trials in Experiment 2 and −60 ms offset trials in Experiment 3. This is because those offsets are largely deviated from the offsets used in the corresponding Conditioning sessions and might attenuate an effect of temporal conditioning on weight perception. In addition to time offset, magnitude of load force was also selected randomly. The magnitude values are listed in Table 2 with their appearance frequency within a single set. Note that the appearance frequencies listed in the table are for each time offset. Therefore, the total number of weight judgment trials in a single set was 69 (23 trials × 3 time offsets) in Experiment 1, and 46 (23 trials × 2 time offsets) in Experiments 2 and 3.

**Table 2:** **Load force values and frequencies in the Weight Perception Test session**.

Force magnitude [N] (appearance frequency for each time offset within a single set)								
2.94 (1)	3.185 (3)	3.43 (3)	3.675 (3)	3.92 (3)	4.165 (3)	4.41 (3)	4.655 (3)	4.9 (1)

In the weight judgment trials, participants were asked not to overcorrect for the reference force. Although we asked them to prevent their hands from moving excessively downward upon force exertion, we also instructed them that it was not necessary to return their hands to the initial position. The instructions for the ball-catching task were the same as those for the Conditioning session. After catching the ball, participants were required to judge the heaviness of the load force compared to the reference force. Participants reported which force they perceived as heavier by pressing the left or right button of a computer mouse.

### 2.4 Data analysis

All experiment data were analyzed using Matlab and its Statistical Toolbox (Mathworks, MA, USA).

#### 2.4.1 Perceptual judgment analysis

For the Simultaneity Test session, the judgment of participants was modeled to a psychometric curve. Using the Matlab function “glmfit,” the probability of judging “load force preceding visual contact” was fitted with a sigmoid function,

(2) $$p\left(\text{judging force first}\right)=\frac{1}{1+\exp\left(\theta_{0}+\theta_{1}\Delta t\right)}\;,$$

where Δ*t* is time offset, and θ_0_ and θ_1_ are the regression coefficients. A psychometric curve was made for each participant using their individual judgments. A group psychometric curve was also made from the judgments across all participants. The point of subjective simultaneity (PSS), where Δ*t* gives *p* = 0.5, can be calculated as $\text{PSS}=-\frac{\theta_{0}}{\theta_{1}}$.

For the Weight Perception Test session, the participants’ judgments were again modeled to a psychometric curve. The probability of judging that “the ball was heavier than the reference force” was fitted with a sigmoid function,

(3) $$p\left(\text{judging ball heavier}\right)=\frac{1}{1+\exp\left(\phi_{0}+\phi_{1}\Delta F\right)}\;,$$

where Δ*F* is the percent difference in load force magnitude compared to that of the reference force and takes a negative value when the load force is comparatively smaller. Both individual and group psychometric curves were computed for each time offset used in each experiment. The point of subjective equality (PSE), where Δ*F* gives *p* = 0.5, can be calculated as $\text{PSE}=-\frac{\phi_{0}}{\phi_{1}}$.

#### 2.4.2 Movement kinematics analysis

To analyze kinematics of the hand motion during ball-catching, we considered the grip position in the vertical direction as the hand position. The positional data were digitally low-pass filtered at 20 Hz using fourth-order Butterworth filter. We calculated a baseline hand position for each trial by averaging hand position within a period from 200 ms before to the moment the ball started falling. The baseline position was then subtracted from the position data. In addition, hand acceleration was computed by numerically differentiating the position data twice.

From the hand acceleration data, we also determined the timing when a ball-catching motion was initiated relative to the timing of visual contact. Here, let us call this timing as motion initiation timing. To acquire the motion initiation timing in each trial, a baseline acceleration variance for each trial was acquired by computing the standard deviation of hand acceleration during the baseline period (i.e., from 200 ms before to the moment the ball started falling). The motion initiation timing was then determined as the first point that the hand acceleration exceeded some threshold value. In this study, we set the threshold as 20 times the baseline acceleration variance. We confirmed that main results did not change by setting the threshold from 10 to 30 times the baseline acceleration variance.

## 3 Results

### 3.1 Movement kinematics

To investigate how movement kinematics changed as magnitude or timing of load force changed, we analyzed the hand trajectories during ball-catching motion in Weight Perception Test session of Experiment 1. For each participant, the hand position data were aligned at the time of load force exertion, and averaged within the trials with the same load force magnitude and the same time offset. We consider the average hand trajectory of 0 ms offset trials with 3.92 N load force as a reference trajectory. This is because those values were consistently used in Conditioning session of Experiment 1. The difference between the reference trajectory and other average trajectory was assessed by averaging hand position difference from 200 ms before to 200 ms after the load force timing.

**Figure 3:**
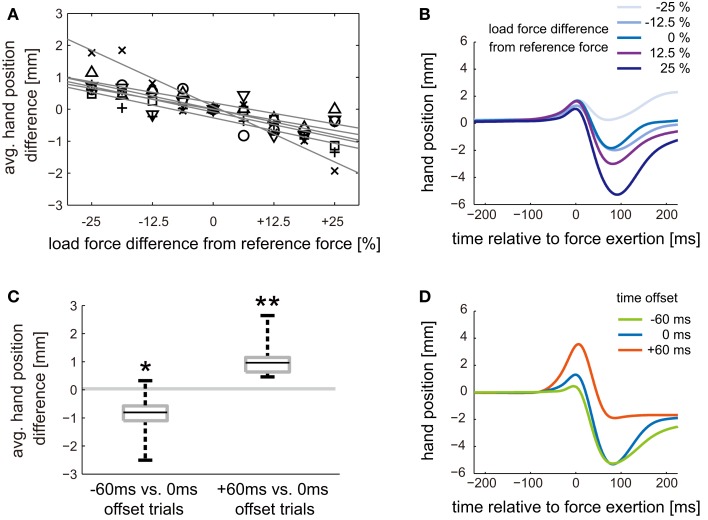
**Movement kinematics difference in Experiment 1**. **(A)** Trajectory difference caused by difference in load force magnitude. Hand trajectory data are aligned at the time of load force exertion and averaged within the trials with the same combination of time offset and load force magnitude. The difference between average hand trajectory of 0 ms offset with 3.92 N force and that of 0 ms offset with other force magnitude was assessed by averaging hand position difference from 200 ms before to 200 ms after the load force exertion. Shape of markers represents participant. The gray solid lines are linear regressions of each marker. The negative value in trajectory difference means that the hand was on average below the reference trajectory. **(B)** Typical hand trajectories for different load force magnitude. Average hand trajectories of 0 ms offset trials with each of five different load force magnitude are aligned at the time of load force exertion. The data shown in the figure are the ones of a typical participant. **(C)** Trajectory difference caused by difference in time offset. The average hand position differences between average hand trajectory of 0 ms offset with 3.92 N force and those of −60 and +60 ms with 3.92 N force offsets are computed for each participant and plotted as box plots. Asterisks denote that the difference was significantly greater or less than zero: *P < 0.05, **P < 0.01. **(D)** Typical hand trajectories for different time offsets. Average hand trajectories of −60, 0, and +60 ms offset trials with 3.92 N force are aligned at the time of load force exertion. The data shown in the figure are the ones of another typical participant.

Figure 3A shows the average hand position difference caused by the difference in load force magnitude. All participants showed negative correlation between load force magnitude and the average hand position difference. Mean correlation coefficient between the two variables was −0.89 (SD: 0.07) across participants. This result indicates that the hand moved more downward as the magnitude of load force became larger. Figure 3B shows the time series data of hand position of a typical participant. The average hand trajectories of 0 ms offset trials with each of five different load force magnitude are aligned at the time of load force exertion. The similar upward movements before load force exertion can be seen in all trajectories. On the other hand, the hand moved differently after load force was applied to the hand. The hand tended to move further downward as the force magnitude became larger.

The trajectory difference induced by the difference in time offset is shown in Figures 3C,D. The average hand position difference between −60 and 0 ms offset trials was −0.91 (SD: 0.83) mm averaged across participants (Figure 3C). This difference was significantly less than zero (*t*(5) = −2.4; *P* = 0.029). On the other hand, the average hand position difference between +60 and 0 ms offset trials was 1.14 (SD: 0.72) mm averaged across participants. This difference was significantly greater than zero (*t*(5) = 3.5; *P* = 0.0083). We can say that the hand moved more downward in −60 ms offset trials compared to 0 ms offset trials. On the other hand, the hand moved more upward in +60 ms offset trials. Figure 3D shows the time series data of hand position of another typical participant. The average hand trajectories of −60, 0, and +60 ms offset trials with 3.92 N load force are aligned at the time of load force exertion. We can see that the difference in time offset caused difference in upward movements before load force exertion. The hand started moving earlier, relative to the timing of load force exertion, in +60 ms offset trials compared to 0 ms offset trials. As the result, the hand moved more upward before the load force was applied to the hand. In contrast, the upward movement became smaller in −60 ms offset trials. This is because the hand set off later than 0 ms offset trials. Meanwhile, since the load force magnitude was the same, the amounts of downward movements from positive to negative peak positions in each of the three trajectories did not differ so much with each other.

### 3.2 Motion initiation timing

**Figure 4:**
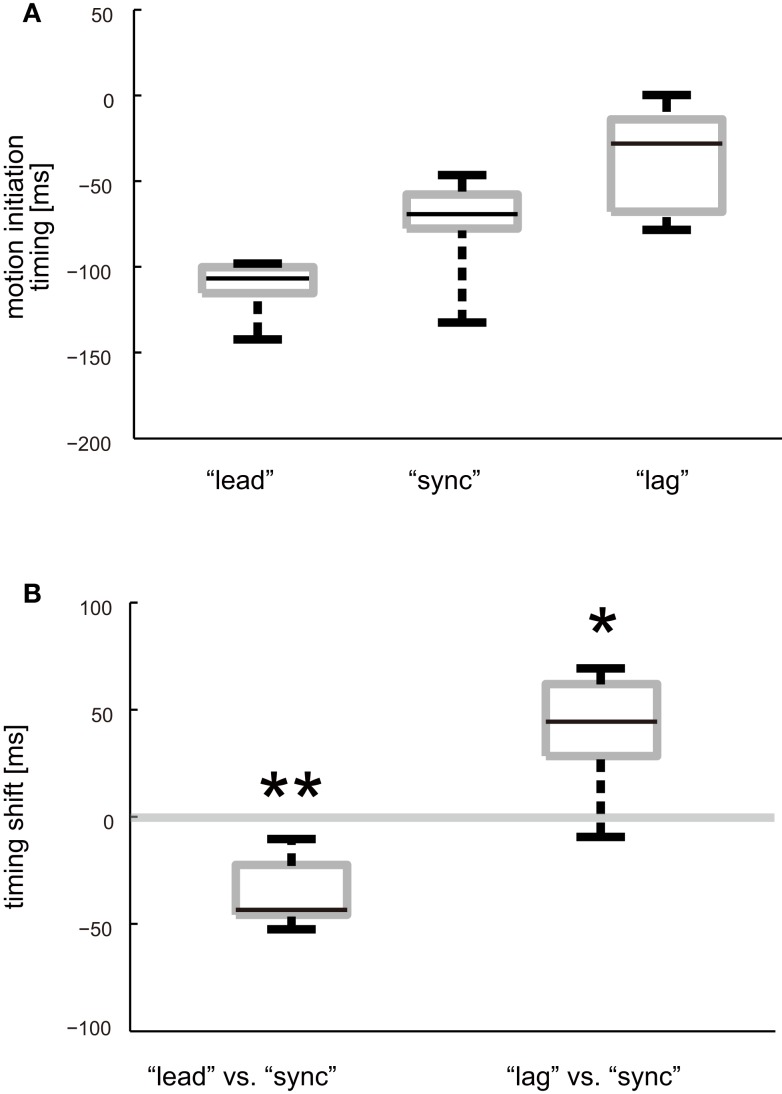
**Timing of ball-catching motion initiation**. **(A)** Box plots of average motion initiation timing after “lead,” “sync,” and “lag” conditioning. Average motion initiation timing was calculated for each participant and each experiment. **(B)** Box plots of shifts in average motion initiation timing after different types of offset conditioning. Amount of shift for each participant was calculated by subtracting average motion initiation timing in the Weight Perception Test session of Experiment 1 (after “sync” conditioning) from those of Experiment 2 (after “lead” conditioning) and 3 (after “lag” conditioning). Asterisks denote that the timing shift was significantly greater or less than zero: *P < 0.05, **P < 0.01.

The timing of motion initiation relative to visual contact was investigated to reveal how participants changed the timing of their catching motion after “sync,” “lead,” and “lag” conditionings. The average motion initiation timing after each conditioning was acquired by taking the average of the trials in the Weight Perception Test session. We used the trials with the same time offset as that in the corresponding Conditioning session, i.e., 0, −60, and +60 ms offset trials for Experiment 1, 2, and 3, respectively. Figure 4A shows box plots of average motion initiation timings. The average value across participants were −112.0 (SD: 15.0) ms, −75.3 (SD: 27.3) ms, and −35.9 (SD: 28.8) ms for “lead,” “sync,” and “lag” conditionings, respectively.

We also analyzed the amount of the shifts in the motion initiation timings after “lead” and “lag” conditioning from that after “sync” conditioning (Figure 4B). The average timing shifts from “sync” to “lead” conditioning was −36.2 (SD: 16.4) ms across participants. This shift was significantly less than zero (*t*(5) = −5.4; *P* = 0.0015). On the other hand, the average timing shifts from “sync” to “lag” conditioning was 39.8 (SD: 29.2) ms across participants. This shift was significantly greater than zero (*t*(5) = 3.34; *P* = 0.010). It can be said that the participants had adjusted their motion initiation timing to match the persistently exposed time offset between visual contact and load force exertion.

### 3.3 Simultaneity shift

**Figure 5:**
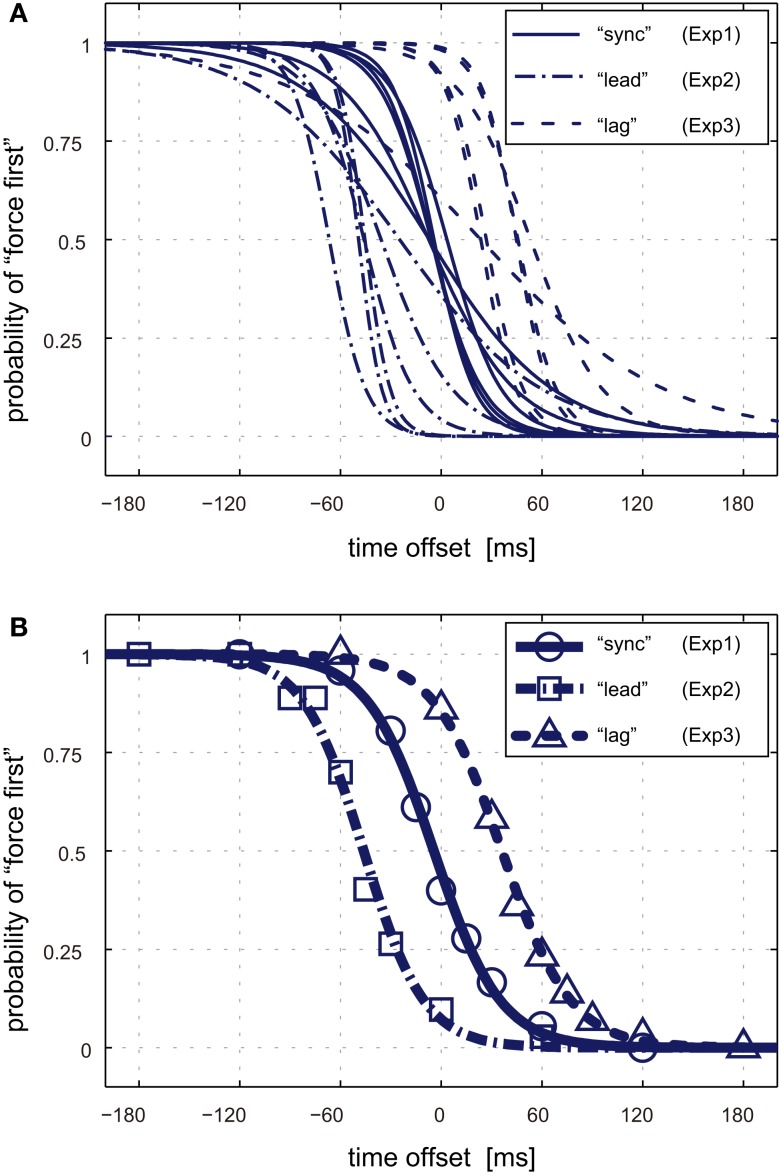
**Psychometric curves for temporal simultaneity**. **(A)** Psychometric curves of each participant after “sync” (solid lines), “lead” (dash-dotted lines), and “lag” (dotted lines) conditioning. The horizontal axis represents time offset between load force exertion and visual contact. Negative or positive offsets indicate that force exertion preceded or followed visual contact, respectively. The vertical axis represents probability that the participants judged that load force was applied before visual contact. **(B)** Group-average psychometric curves after the three types of time offset conditioning. Circles, squares, and triangles represent the group-average probability for the “sync,” “lead,” and “lag” conditioning, respectively.

For the Simultaneity Test sessions, we analyzed the temporal order of the visual contact and the load force events. Psychometric curves representing the probability that the participants judged that load force preceded visual contact were acquired by applying a logistic regression model to the participants’ judgments. Psychometric curves for each participant and each conditioning type are shown in Figure 5A. Note that the sign of time offset is positive when the load force was applied after visual contact. The subjective simultaneity of the two events was evaluated by the PSS of the psychometric curves [see equation (2)]. The average PSS across participants were −44.7 (SD: 14.0) ms, −4.5 (SD: 4.0) ms, and 36.5 (SD: 13.1) ms after the “lead,” “sync,” and “lag” conditionings, respectively. The group-average psychometric curves are also shown in Figure 5B. The curves for “lead” and “lag” conditioning were clearly shifted leftward and rightward, respectively, in comparison to “sync” conditioning. The PSS shifts for “lead” conditioning with respect to “sync” conditioning were −40.1 (SD: 15.2) ms averaged across participants. This was significantly less than zero according to a one-sided *t* test (*t*(5) = −6.47; *P* = 0.0007). On the other hand, the PSS shifts for “lag” conditioning with respect to “sync” conditioning were 40.8 (SD: 15.4) ms averaged across participants. This was significantly greater than zero (*t*(5) = 6.41; *P* = 0.0006). Therefore, it can be said that the PSS shifted toward the direction of persistently exposed time offset between visual contact and load force exertion.

### 3.4 Weight perception after “sync” conditioning

**Figure 6:**
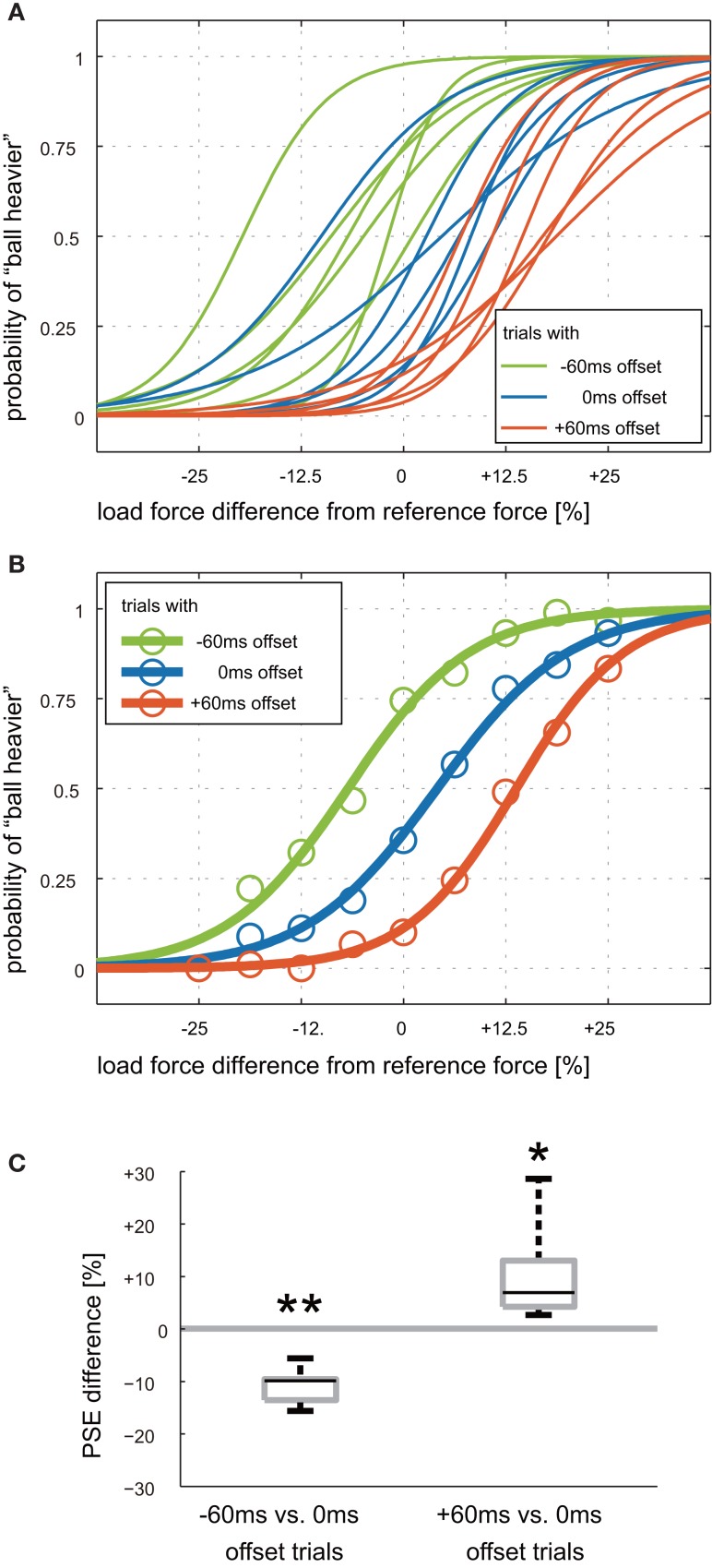
**Psychometric curves for weight judgment after “sync” conditioning**. **(A)** Psychometric curves for each participant. Green, blue, and red colors represents −60, 0, and +60 ms time offsets in weight judgment trials. Offset values are positive in the case where force was applied after visual contact. The horizontal axis represents percent difference in load force magnitude from the reference force. **(B)** Group-average psychometric curves. Circles represent group-average of the probability of judging that the load force was heavier than the reference force. **(C)** Box plots of the difference in point of subjective equality (PSE). The left box represents the difference in PSEs for 0 and −60 ms offset trials. The right box represents the difference in PSEs for 0 and +60 ms offset trials. The whiskers correspond to minimum and maximum values. Asterisks denote that the difference in PSE is significantly greater or less than zero: *P < 0.05, **P < 0.01.

In Experiment 1, participants were conditioned to 0 ms time offset where load force exertion was synchronized with visual contact. The Weight Perception Test session was then conducted to examine how unpredictably induced time offsets affect perception of load force magnitude. Three different offsets (−60, 0, +60 ms) were used in this session. Figure 6A shows each participant’s psychometric curves for each offset. The curves represent, as functions of the percent difference in load force magnitude compared to that of the reference force, the probability of judging that the load force was heavier than the reference force. Although psychometric curves differed from participant to participant, they moved toward the right as offset increased from −60 ms to +60 ms. This tendency can be clearly seen in the plot of group-average psychometric curves (Figure 6B). The psychometric curve shifts indicate that the same magnitude of load force was perceived differently as time offset changed. For example, when the load force magnitude was the same as that of the reference force (0% on the horizontal axis), the probability that participants perceived the ball heavier became larger as the offset became negative (i.e., the load force preceded). The same tendency can be seen for all other magnitudes.

The difference in perceived heaviness can also be evaluated by the difference in PSE of the psychometric curves [see equation (3)]. The PSE indicates the magnitude of load force perceived to be the same as that of the reference force. The average PSE across participants were −6.9 (SD: 7.2) %, 3.9 (SD: 7.4) %, and 14.3 (SD: 4.5) % for −60, 0, +60 ms offset trials, respectively. Note that the smaller the PSE, the heavier the load force perceived. The PSE shifts for each participant are shown as box plots in Figure 6C. The average PSE shifts from 0 to −60 ms offset trials was −10.7 (SD: 3.5) % and was significantly less than zero (*t*(5) = −7.53; *P* = 0.00003). The average PSE shifts from 0 to +60 ms offset trials was 10.4 (SD: 9.7) %. This shift was significantly greater than zero (*t*(5) = 2.64; *P* = 0.023). It can be interpreted that the load force exerted earlier than visual contact was perceived as heavier than that exerted at the same time as visual contact, and when the load force was exerted later than visual contact, it was perceived as lighter.

### 3.5 Weight perception after “lead” and “lag” conditioning

The Weight Perception Test sessions in Experiments 2 and 3 were conducted to examine how participants’ weight perceptions were affected after conditioning to negative or positive time offsets. In the Conditioning session in Experiment 2 (“lead” conditioning), participants performed 80 ball-catching trials in which load force preceded visual contact by 60 ms. For the Conditioning session in Experiment 3 (“lag” conditioning), load force followed visual contact by 60 ms. Weight judgments made in Experiments 2 and 3 were compared to those of Experiment 1 (“sync” conditioning) shown in Figure 6B.

**Figure 7:**
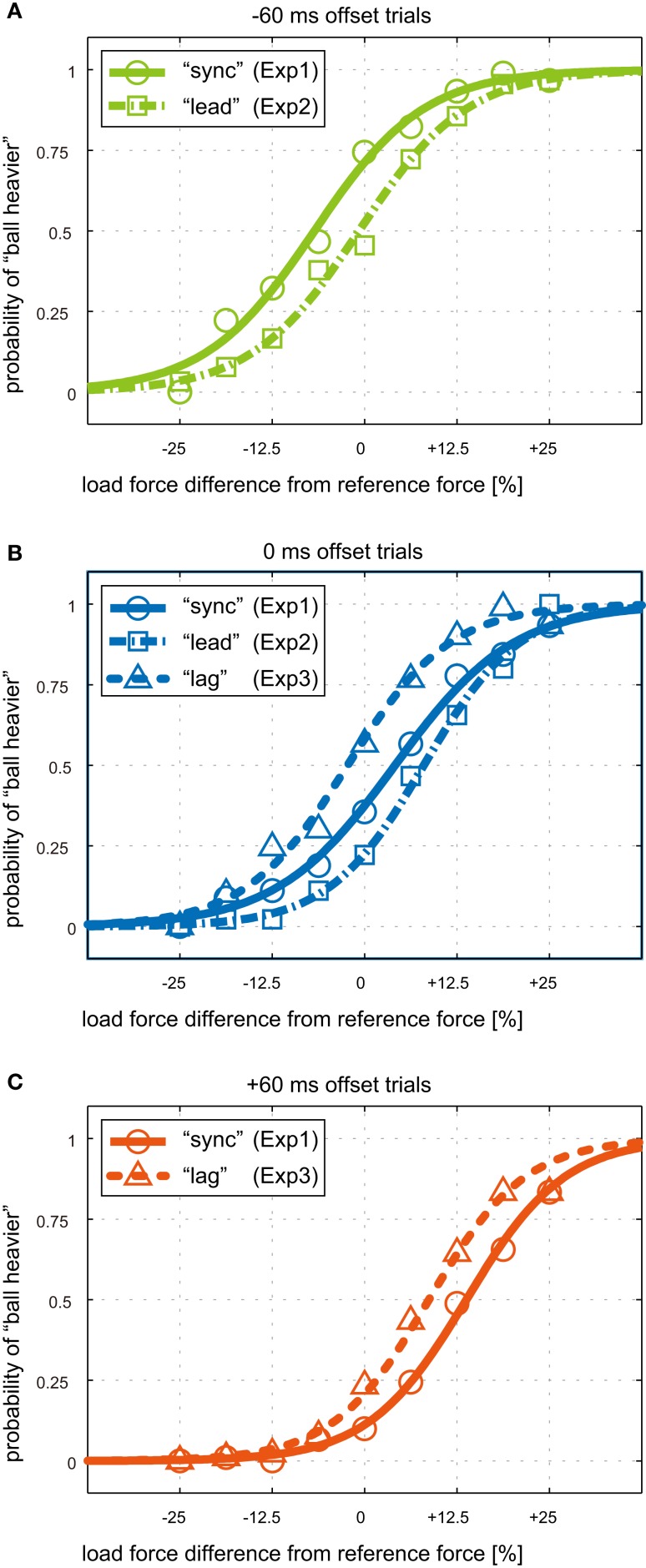
**Group-average psychometric curves for weight judgment in the three experiments**. Psychometric curves for **(A)**−60 ms offset trials, **(B)** 0 ms offset trials, and **(C)** +60 ms offset trials in the Weight Perception Test sessions. Offset values are positive in the case where force was applied after visual contact. The horizontal axes represent the percent difference in load force magnitude from the reference force. Green, blue, and red colors represents −60, 0, and +60 ms time offsets. Solid, dash-dotted, and dotted lines correspond to Weight Perception Test sessions after “sync,” “lead,” and “lag” conditioning, respectively.

Figure 7 shows group-average psychometric curves for the weight judgments made at −60 ms (Figure 7A), 0 ms (Figure 7B), and +60 ms (Figure 7C) offsets trials, respectively. For both of the −60 and 0 ms offset trials, curves for “lead” conditioning shifted toward the right compared to those for “sync” conditioning (Figures 7A,B). Note that the rightward psychometric curve shifts indicate that the participants perceived the ball’s weight to be lighter in weight judgment trials after “lead” conditioning compared to those after “sync” conditioning, even though actual magnitude and timing of force exertion were the same. Curves shifted toward the left in “lag” conditioning compared to “sync” conditioning (Figures 7B,C), showing that the ball’s weight was perceived heavier in weight judgment trials after the “lag” conditioning.

**Figure 8:**
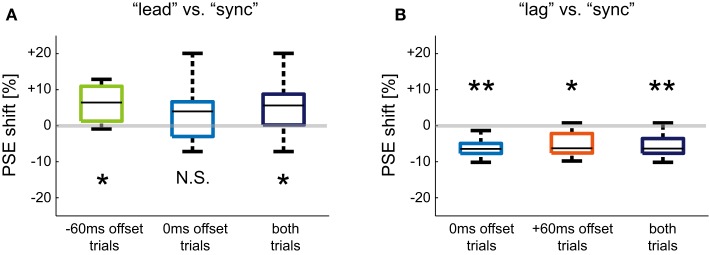
**Shift in PSE after different types of offset conditioning**. **(A)** Box plots of PSE shift for “lead” vs. “sync” conditioning. **(B)** Box plots of PSE shift for “lag” vs. “sync” conditioning. The PSE shifts are plotted separately with respect to time offsets used in the Weight Perception Test sessions. A set of PSE shifts for both offsets is also plotted. Asterisks denote that the PSE shift was significantly greater or less than zero: *P < 0.05, **P < 0.01.

Change in perceived weight was evaluated by PSE shifts in the psychometric curves of each participant. The amounts of PSE shift for “lead” vs. “sync” conditioning are shown as box plots in Figure 8A with respect to the two offset values. A box plot of the set of PSE shifts for both offsets is also shown in the figure. The average PSE shifts across participants for −60 ms offset trials and 0 ms offset trials were 6.2 (SD: 5.3) % and 4.1 (SD: 9.4) %, respectively. The average PSE shifts across participants and the two offset trials was 5.1 (SD: 7.4) %. The PSE shift for −60 ms offset trials was significantly greater than zero (*t*(5) = 2.84; *P* = 0.018). Although the PSE shift for 0 ms offset trials was not significantly greater than zero (*t*(5) = 1.07; *P* = 0.167), the PSE shift for the set of offset trials was significantly greater than zero (*t*(5) = 2.42; *P* = 0.017). Figure 8B shows the amount of PSE shift for “lag” vs. “sync” conditioning. The average PSE shifts across participants were −6.2 (SD: 3.0)% and −5.2 (SD: 3.9) %, for 0 and +60 ms offset trials, respectively. Those shifts were significantly less than zero (*t*(5) = −5.12; *P* = 0.002 and *t*(5) = −3.32; *P* = 0.011). The average PSE shifts across participants and the two offset trials was −5.7 (SD: 3.3) % and significantly less than zero (*t*(5) = −5.12; *P* = 0.00005). These results indicate that the participants’ ball weight perception was changed by being conditioned to time offset between load force exertion and visual contact. The participants began to perceive the ball’s weight as lighter after “lead” conditioning and heavier after “lag” conditioning.

### 3.6 Relationship between PSE and PSS

**Figure 9:**
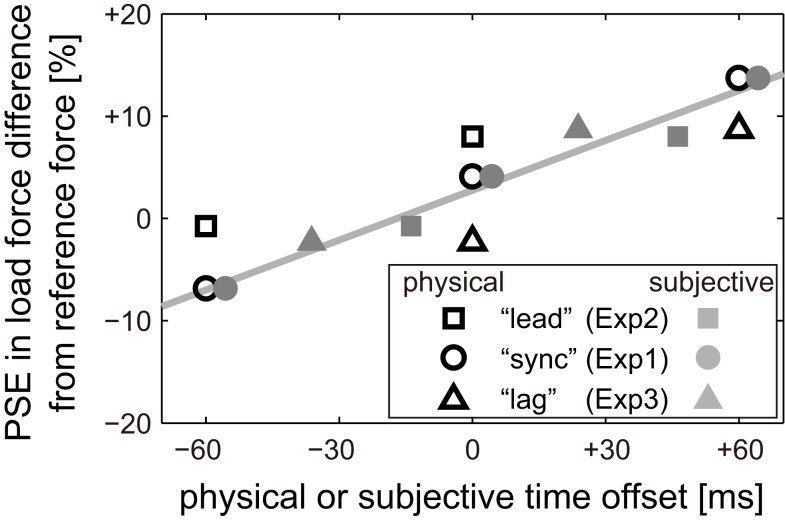
**Relationship between perceived weight and physical or subjective time offset**. PSEs of group-average psychometric curves for weight judgments in all experiments are plotted with respect to actual (physical) time offsets as black unfilled marks. PSEs are also plotted as gray filled marks with respect to subjective time offsets (physical offset minus PSS of group-average psychometric curves for temporal simultaneity). Shapes of the marks represent the Conditioning sessions. The gray solid line is a linear regression of the gray marks.

The results of the weight judgment trials in Experiment 1 revealed that the weight of the falling ball was perceived differently by introducing time offsets between load force exertion and visual contact (Figure 6). In Experiment 2 and 3, we also found that the weight of the ball was perceived differently after “lead” and “lag” conditioning, even though the time offsets were the same as those used in the weight judgment trials in Experiment 1 (Figure 7). Therefore, the perceived weight illusion observed in Experiment 1 seems not to be related to actual physical time offset between visual contact and load force exertion. Rather, the illusion in weight perception seems to be connected to the participants’ subjective perception of time offset. This subjective time offset is thought to be modified by shifts in PSS after “lead” and “lag” conditioning. To show how perceived weight is related to physical or subjective time offset, the PSEs of group-average psychometric curves shown in Figure 7 are plotted with respect to their corresponding physical or subjective offsets (Figure 9). Here the subjective time offsets were calculated by subtracting the PSS of the group-average psychometric curve in each experiment from the physical time offsets used in the Weight Perception Test session in each experiment. We can see that the same physical offset yielded different PSE values (black open marks). On the other hand, PSE values plotted with respect to subjective offset (gray filled marks) increased approximately linearly with subjective offset value. The correlation coefficient of PSS and subjective time lag was 0.98.

### 3.7 Relationship between PSE and estimation error in force exertion timing

In addition to the subjective perception of time offset, the estimation of force exertion timing in sensorimotor system also seems to be modified after “lead” and “lag” conditioning. Here we analyze the relationship between perceived weight and estimation error in force exertion timing. Although we cannot directly measure the estimation of force exertion timing, we can infer it from the motion initiation timing relative to visual contact. Let us assume that ball-catching motions are initiated some fixed second in advance of the estimated timing of force exertion. This assumption is supported by other experiments in which the timings of muscle activity and catching motion were found to be consistently initiated a few hundred millisecond before the ball contacts the hand (Lacquaniti and Maioli, 1989; Zago et al., 2004; Hong et al., 2005; Kambara et al., 2011; Kawase et al., 2012). We also assume that the margin between the motion initiation timing and the estimated timing varied among participants, but did not change within a single participant in the three experiments. According to these assumptions, we inferred changes in the estimation of force timing by analyzing changes in the motion initiation timing relative to visual contact.

**Figure 10:**
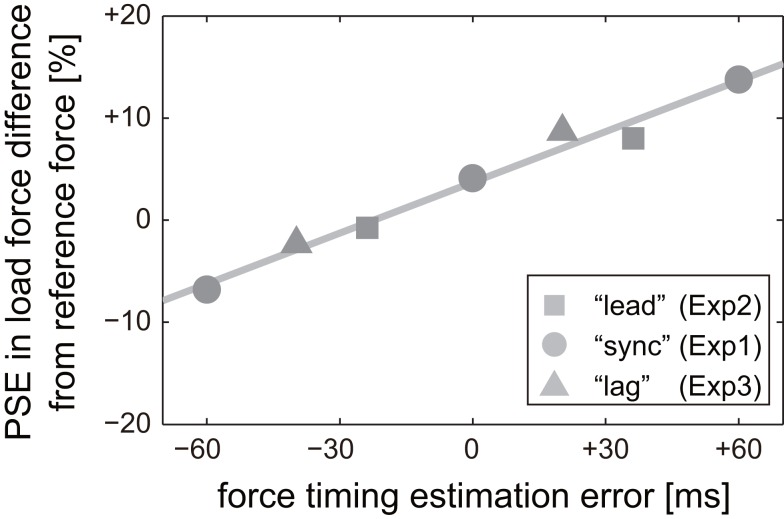
**Relationship between perceived weight and estimation error in load force exertion timing**. PSEs of group-average psychometric curves for weight judgments in all experiments are plotted with respect to the estimation error in load force exertion timing. The estimation error for α ms offset trials in Experiment β was calculated by subtracting group-average motion initiation timing in Experiment β from the sum of α ms offset and group-average motion initiation timing in Experiment1.

Figure 10 plots the PSEs of group-average psychometric curves shown in Figure 7 against the estimation error in load force exertion timing. Note that we assumed that there was no estimation error in 0 ms offset trials after “sync” conditioning (Experiment 1). The estimation error was then calculated by subtracting group-average motion initiation timing in each experiment from the sum of time offset and group-average motion initiation timing in Experiment 1. For example, the estimation error in +60 ms offset trials in Experiment 3 was 20.1 (=60 + (−75.3) − (−35.9)), where the values of −75.3 and −35.9 ms correspond to group-average motion initiation timings in Experiment 1 and 3, respectively (values of group-average motion initiation timing in each experiment is described in Section 2). We can see that the PSE values increased almost linearly with the estimation error. The correlation coefficient of PSE and the estimation error was 0.99.

## 4 Discussion

In this study, we investigated the effect of force exertion timing on perceived magnitude of force. We conducted three experiments with a virtual reality system in which the load force of a falling ball was applied at various timings relative to visual contact with the hand in the display. In Experiment 1 (“sync” conditioning), we tested how force exertion timing influenced perception of the ball’s weight. We found that the ball was perceived to be heavier when the load force was applied before visual contact. In contrast, the ball was perceived to be lighter when the load force was applied after visual contact. The results exhibited the illusion in which the same magnitude of load force was perceived differently as the timing of force exertion changed. In Experiments 2 (“lead” conditioning) and 3 (“lag” conditioning), we tested how this illusion would be modified by persistent pre-exposure to time offsets between load force exertion and visual contact. We first found that the PSS in regards to temporal order of load force exertion and visual contact shifted toward the conditioned offset. The timings of ball-catching motion initiation relative to visual contact were also shifted in the direction that the time difference between load force exertion and motion initiation became close to that after “sync” conditioning, where load force was applied at the time of visual contact. Next, we found that the weight experienced at physically identical time offset relative to visual contact was perceived lighter after the participants had undergone “lead” conditioning, where load force preceded visual contact by 60 ms, and heavier after “lag” conditioning, where load force was applied 60 ms after visual contact. We confirmed that the amount of difference in perceived weight could be linearized with respect to both the subjective time offset related to the shift in PSS and the estimation error in load force exertion timing inferred from the motion initiation timing. These results suggest that the observed illusion may not originate from physical time offset between force exertion and visual contact. Rather, the illusion may originate from the perceptual simultaneity between force exertion and visual contact and/or the estimation error in force exertion timing in sensorimotor system.

Since many types of force and weight illusions, including the size-weight-illusion, have became widely known, several hypotheses have been proposed to explain them. The mismatch hypothesis claims that the illusion originates from a mismatch between an internal sensorimotor prediction and actual sensory feedback (Ross, 1969; Davis and Roberts, 1976; Kim et al., 2002; Koike et al., 2006; Diedrichsen et al., 2007). However, it has also been suggested that the illusion is caused by a high-level cognitive and perceptual mismatch of expected weight and sensed weight that is independent from the sensorimotor system (Flanagan and Beltzner, 2000; Flanagan et al., 2008; Brayanov and Smith, 2010). Although we still lack a definitive explanation of why the illusions occur (Ernst, 2009), we can say that the illusions, albeit in the sensorimotor or perceptual system, coincide with a mismatch between expectation and experience. In this study, we showed that the changes in the temporal factors related to both of the sensorimotor and perceptual systems had been synchronized with the changes in weight perception. In the following sections, we discuss the effects of each system on the weight perception.

### 4.1 Effect of estimation error in sensorimotor system on weight perception

A linear relationship between perceived weight of the ball and estimation error in the timing of load force exertion (Figure 10) indicates a possibility that the estimation error made by the sensorimotor system caused the difference in perceived heaviness. The estimation of TTC, that is, the time remaining before contact, is required to generate anticipatory motion for the successful catch of a falling ball. The motor command for the catching motion seems to be sent to the muscles in advance of the estimated timing of contact (Lacquaniti and Maioli, 1989; Hong et al., 2005; Kambara et al., 2011; Kawase et al., 2012). It has been proposed that sensorimotor control system is utilizing internal forward model of the body and world to predict sensory signals before actual sensory feedback is acquired (Desmurget and Grafton, 2000; Wolpert and Ghahramani, 2000; Shadmehr et al., 2010). The misperception of the ball’s weight might have originated from the mismatch between the internally predicted motor outcome and actually sensed outcome caused by the incorrect TTC estimate.

In Experiment 1, it is reasonable that the timing of load force exertion was estimated based on 0 ms time offset, which is the time offset persistently experienced in “sync” conditioning. When the actual time offset was −60 ms, then, participants’ hand moved further downward than the internally predicted trajectory according to 0 ms time offset (see Figures 3C,D). In contrast, unexpected upward movement seemed to be observed in +60 ms time offset trials where the load force was applied later than the estimated timing. Note that, when we hold an object, our hand moves downward or upward when the weight of the object is heavier or lighter than expected (see Figures 3A,B). It has been reported that deafferented patients can discriminate the weight of two objects almost as well as normal subjects when they are allowed to observe their movements visually (Rothwell et al., 1982; Cole and Sedgwick, 1992). Those studies indicate that observing the mismatch between internally predicted and actual motor outcomes strongly affects a decision of the heaviness of held objects. Therefore, the unexpected downward and upward hand motion caused by the error in TTC estimation may be connected with the same motion caused by misestimating the weight of the ball.

### 4.2 Effect of subjective simultaneity in perceptual system on weight perception

**Figure 11:**
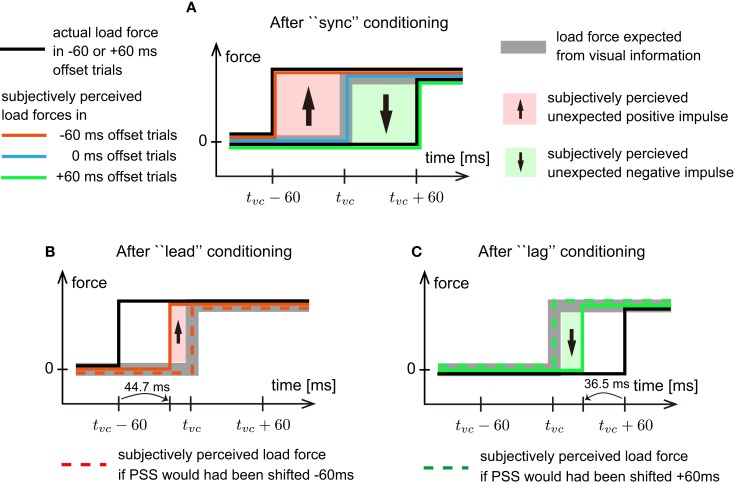
**Weight perception illusion occurred in perceptual system**. **(A)** Schematic model explaining weight perception difference caused by time offset difference after “sync” conditioning. Black solid lines represent time load force actually applied to the hand in −60 or +60 ms offset trials. Gray bold line is the load force expected from visual information. Red, blue, and green lines trace subjectively perceived load forces in −60, 0, and +60 ms offset trials, respectively. Note that perceived load forces are coincided with subjectively perceived timings of load force exertion. An area colored by light red with an up-pointing arrow represents unexpected positive force impulse. Here positive force impulse means that unexpected force was perceived during that period. An area colored by light green with a down-pointing arrow represents unexpected negative force impulse. Here negative force impulse means that expected force was not perceived during that period. *t_vc_* is the time of visual contact. **(B)** Schematic model for weight perception in −60 ms offset trials after “lead” conditioning. A red dashed line represents the subjectively perceived load force assuming −60 ms shift in PSS. Note that the group-average PSS after “lead” conditioning was −44.7 ms. **(C)** Schematic model for weight perception in +60 ms offset trials after “lag” conditioning. A green dashed line represents the subjectively perceived load force assuming +60 ms shift in PSS. Note that the group-average PSS after “lag” conditioning was 36.5 ms.

In addition to the estimation error in sensorimotor system, subjectively perceived time offset also showed a linear relationship to the perceived weight of the ball. Here we discuss an effect of subjective simultaneity in the perceptual system on weight perception. First, we consider the difference in weight perception elicited by different time offsets in Experiment 1 (after “sync” conditioning). Let us assume that expectation of the load force in the perceptual system always synchronized with actual visual information (Figure 11). In other word, the load force was not expected to be applied before the ball contact the hand in the display, but was expected to be applied after the visual contact. In −60 ms offset trials, the perceptual system starts experiencing, from 60 ms before to the time of visual contact, the force not expected from the visual information. Then, the mismatch occurred between expected and experienced force until the visual contact. It is possible that the unexpected force impulse sensed in the perceptual system made the ball to be perceived heavier in −60 ms offset trials (Figure 11A). In +60 ms offset trials, the perceptual system do not experience visually expected force until 60 ms after the visual contact. The absence of expected force might have made participants to perceived the ball lighter.

Next, let us consider how “lead” and “lag” conditionings changed the weight perception against the same visual and haptic stimuli. Common with previous studies testing subjective simultaneity between voluntarily generated motor events and visual stimuli (Cunningham et al., 2001; Haggard et al., 2002; Stetson et al., 2006; Heron et al., 2009; Sugano et al., 2010; Tanaka et al., 2011), the PSS shifted to reduce subjectively perceived time offset in the Conditioning sessions for “lead” and “lag.” In addition, it has been suggested that the change in PSS is caused by a shift in interpretation about the timing when motor events occurred with respect to visual stimuli (Sugano et al., 2010). According to this hypothesis, in our study, the subjectively perceived timing of force exertion relative to the timing of visual contact seemed to be delayed after “lead” conditioning and be advanced after “lag” conditioning (Figures 11B,C). In −60 ms offset trials after “lead” conditioning, the duration that the perceptual system is subjectively perceiving unexpected load force becomes shorter compared to that in −60 ms offset trials after “sync” conditioning (see the difference in the areas colored with light red in Figures 11A,B). The reduction in the subjectively perceived unexpected force impulse might be the reason why participants perceived the ball as lighter after “lead” conditioning compared to that after “sync” conditioning. Note that if PSS had been shifted 60 ms after “lead” conditioning, the weight perceived in −60 ms offset trials would have been as same as the weight perceived in 0 ms offset trials in “sync” conditioning. However, the PSS shifted only 40.1 ms on average in our experiment. The difference between the perceived weight in 0 ms offset trials after “sync” conditioning and that in −60 ms offset trials after “lead” conditioning might be caused by incompleteness in the PSS shift. In +60 ms offset trials after “lag” conditioning, on the other hand, the duration that the perceptual system is experiencing the absence of expected force reduces compared to −60 ms offset trials after “sync” conditioning (see the difference in the areas colored with light green in Figures 11A,C). For this reason, the ball might have been perceived as heavier compared to the same offset trials after “sync” conditioning. Finally, if we suppose that weight illusion is caused by mismatch between expectation and experience in perceptual system, it can be said that the different temporal conditionings caused different weight perception not because the expectation of the weight was changed, but because the way perceptual system experience the weight had been changed.

### 4.3 Causality of weight illusion

In our experiment, the change in weight perception was synchronized with the changes in temporal factors in both perceptual and sensorimotor systems. On the other hand, Flanagan et al. (2008) showed that size-weight illusion did synchronize with perceptual mismatching but not with sensorimotor system. In their experiment, subjects practice to lift a set of blocks whose weights vary inversely with volume. During multiday practice in lifting the blocks, size-weight illusion was gradually inverted, that is, the larger block became to be perceived heavier than smaller one. In contrast to gradual change in the weight perception, the lift force, supposed to be related to expected weight in the sensorimotor system, rapidly changed to match the inverted object weights within ten lifts. From these results, Flanagan et al. (2008) concluded that the illusion in weight perception was caused by the mismatch in perceptual system. Unlike the experiment in Flanagan et al. (2008), weight perception had rapidly changed after experiencing tens of ball-catching trials in our experiment. The reason of the difference in the latency of perceptual change seems to be coming from the difference in the factors modified in perceptual system. In Flanagan et al. (2008), expected weight itself was changed. In our experiment, on the other hand, the subjective temporal simultaneity between visual and haptic stimuli was changed. The temporal simultaneity seems to have nothing to do with expected weight in the perceptual system. However, as described before, it has possibility to change the way perceptual system experience the weight. Therefore, it is possible that quick change in perceptual system induced quick change in weight perception.

In our experiment, we could not observe the difference in the rate of changes in the temporal factors in perceptual and sensorimotor systems. For this reason, it is difficult to conclude whether the illusion observed in this study was related to the temporal mismatching in perceptual system or that in sensorimotor system. Further experiments seem to be needed to discriminate the two possibilities. For example, conducting the experiment with supporting the hand will prevent the mismatching in sensorimotor system and will be useful to investigate whether the illusion was driven by sensorimotor system or not.

## Conflict of Interest Statement

The authors declare that the research was conducted in the absence of any commercial or financial relationships that could be construed as a potential conflict of interest.

## References

[B1] Akahane K. Hasegawa S. Koike Y. Sato M. (2006). “A proposal of a high definition haptic rendering for stability and fidelity,” in Proceedings of the 16th International Conference on Artificial Reality and Telexistence, ICAT ’06 (Bunkyo-ku: Virtual Reality Society of Japan), 162–167

[B2] Brayanov J. B. Smith M. A. (2010). Bayesian and “änti-Bayesian” biases in sensory integration for action and perception in the size-weight illusion. J. Neurophysiol. 103, 1518–1531 10.1152/jn.00814.2009 20089821 PMC4422348

[B3] Charpentier A. (1891). Analyse experimentale: de quelques elements de la sensation de poids. Arch. Physiol. Norm. Pathol. 3, 122–135

[B4] Cole J. D. Sedgwick E. M. (1992). The perceptions of force and of movement in a man without large myelinated sensory afferents below the neck. J. Physiol. (Lond.) 449, 503–515 1522522 10.1113/jphysiol.1992.sp019099 PMC1176092

[B5] Cunningham D. W. Billock V. A. Tsou B. H. (2001). Sensorimotor adaptation to violations of temporal contiguity. Psychol. Sci. 12, 532–535 10.1111/1467-9280.00328 11760144

[B6] Davis C. M. Roberts W. (1976). Lifting movements in size-weight illusion. Percept. Psychophys. 20, 33–36 10.3758/BF03198701

[B7] De Camp J. E. (1917). The influence of color on apparent weight: a preliminary study. J. Exp. Psychol. 62, 347–370 10.1037/h0075903

[B8] Desmurget M. Grafton S. (2000). Forward modeling allows feedback control for fast reaching movements. Trends Cogn. Sci. (Regul. Ed.) 4, 423–431 10.1016/S1364-6613(00)01537-0 11058820

[B9] Diedrichsen J. Verstynen T. Hon A. Zhang Y. Ivry R. B. (2007). Illusions of force perception: the role of sensori-motor predictions, visual information, and motor errors. J. Neurophysiol. 97, 3305–3313 10.1152/jn.01076.2006 17344369

[B10] Ernst M. O. (2009). Perceptual learning: inverting the size-weight illusion. Curr. Biol. 19, R23–R25 10.1016/j.cub.2008.10.039 19138585

[B11] Flanagan J. R. Beltzner M. A. (2000). Independence of perceptual and sensorimotor predictions in the size-weight illusion. Nat. Neurosci. 3, 737–741 10.1038/76701 10862708

[B12] Flanagan J. R. Bittner J. P. Johansson R. S. (2008). Experience can change distinct size-weight priors engaged in lifting objects and judging their weights. Curr. Biol. 18, 1742–1747 10.1016/j.cub.2008.09.042 19026545

[B13] Haggard P. Clark S. Kalogeras J. (2002). Voluntary action and conscious awareness. Nat. Neurosci. 5, 382–385 10.1038/nn827 11896397

[B14] Hanson J. V. Heron J. Whitaker D. (2008). Recalibration of perceived time across sensory modalities. Exp. Brain Res. 185, 347–352 10.1007/s00221-008-1282-3 18236035

[B15] Harrar V. Harris L. R. (2008). The effect of exposure to asynchronous audio, visual, and tactile stimulus combinations on the perception of simultaneity. Exp. Brain Res. 186, 517–524 10.1007/s00221-007-1253-0 18183377

[B16] Heron J. Hanson J. V. Whitaker D. (2009). Effect before cause: supramodal recalibration of sensorimotor timing. PLoS ONE 4:e7681 10.1371/journal.pone.0007681 19890383 PMC2766625

[B17] Honda T. Hirashima M. Nozaki D. (2012). Adaptation to visual feedback delay influences visuomotor learning. PLoS ONE 7:e37900 10.1371/journal.pone.0037900 22666408 PMC3364281

[B18] Hong S. Kim J. Sato M. Koike Y. (2005). A research of human’s time-to-contact prediction model for ball catching task (in Japanese). IEICE Trans. Inf. Syst. J88-D2, 1246–1256

[B19] Kambara H. Ohishi K. Koike Y. (2011). Learning strategy in time-to-contact estimation of falling objects. J. Adv. Comput. Intell. Intell. Inform. 15, 972–979

[B20] Kawase T. Ohishi K. Yoneyama K. Kambara H. Koike Y. (2012). Recalibration of time to contact. Rob. Auton. Syst. 60, 742–746 10.1016/j.robot.2011.06.011

[B21] Kim J. Hong S. Sato M. Koike Y. (2002). Examination of the size-weight-illusion by utilizing the SPIDAR (in Japanese). Trans. Virtual Real. Soc. Jpn. 7, 347–354

[B22] Kim S. Ishii M. Koike Y. Sato M. (2000). “Haptic interface with 7DOF using 8 strings: SPIDAR-G,” in Proceedings of the 10th International Conference on Artificial Reality and Tele-existence (ICAT ‘00) (Bunkyo-ku: Virtual Reality Society of Japan), 224–230

[B23] Koike Y. Kim J. Shin D. (2006). Role of stiffness in weight perception. Jpn. Psychol. Res. 48, 174–187 10.1111/j.1468-5884.2006.00316.x

[B24] Lacquaniti F. Maioli C. (1989). The role of preparation in tuning anticipatory and reflex responses during catching. J. Neurosci. 9, 134–148 2913200 10.1523/JNEUROSCI.09-01-00134.1989 PMC6570012

[B25] Ross H. E. (1969). When is a weight not illusory? Q. J. Exp. Psychol. 21, 346–355 10.1080/14640746908400230 5378275

[B26] Rothwell J. C. Traub M. M. Day B. L. Obeso J. A. Thomas P. K. Marsden C. D. (1982). Manual motor performance in a deafferented man. Brain 105(Pt 3), 515–542 10.1093/brain/105.3.515 6286035

[B27] Seashore C. E. (1899). Some psychological statics: 2. The material weight illusion. Univ. Iowa Stud. Psychol. 2, 36–46

[B28] Shadmehr R. Smith M. A. Krakauer J. W. (2010). Error correction, sensory prediction, and adaptation in motor control. Annu. Rev. Neurosci. 33, 89–108 10.1146/annurev-neuro-060909-153135 20367317

[B29] Stetson C. Cui X. Montague P. R. Eagleman D. M. (2006). Motor-sensory recalibration leads to an illusory reversal of action and sensation. Neuron 51, 651–659 10.1016/j.neuron.2006.08.006 16950162

[B30] Sugano Y. Keetels M. Vroomen J. (2010). Adaptation to motor-visual and motor-auditory temporal lags transfer across modalities. Exp. Brain Res. 201, 393–399 10.1007/s00221-009-2047-3 19851760 PMC2832876

[B31] Tanaka H. Homma K. Imamizu H. (2011). Physical delay but not subjective delay determines learning rate in prism adaptation. Exp. Brain Res. 208, 257–268 10.1007/s00221-010-2476-z 21076819

[B32] Wolfe H. K. (1898). Some effects of size on judgments of weight. Psychol. Rev. 5, 25–54 10.1037/h0073342

[B33] Wolpert D. M. Ghahramani Z. (2000). Computational principles of movement neuroscience. Nat. Neurosci. 3, 1212–1217 10.1038/81497 11127840

[B34] Zago M. Bosco G. Maffei V. Iosa M. Ivanenko Y. P. Lacquaniti F. (2004). Internal models of target motion: expected dynamics overrides measured kinematics in timing manual interceptions. J. Neurophysiol. 91, 1620–1634 10.1152/jn.00862.2003 14627663

